# Long-Term Impacts of Post-weaning Social Isolation on Nucleus Accumbens Function

**DOI:** 10.3389/fpsyt.2021.745406

**Published:** 2021-09-20

**Authors:** Cari J. Bendersky, Allison A. Milian, Mason D. Andrus, Ubaldo De La Torre, Deena M. Walker

**Affiliations:** ^1^Department of Behavioral Neuroscience, Oregon Health and Science and University, Portland, OR, United States; ^2^Department of Medicine, Division of Pulmonary and Critical Care Medicine, Oregon Health and Science University, Portland, OR, United States

**Keywords:** adolescence, isolation rearing, reward, dopamine, substance use disorder, gene expression

## Abstract

Adolescence is a period of incredible change, especially within the brain's reward circuitry. Stress, including social isolation, during this time has profound effects on behaviors associated with reward and other neuropsychiatric disorders. Because the Nucleus Accumbens (NAc), is crucial to the integration of rewarding stimuli, the NAc is especially sensitive to disruptions by adolescent social isolation stress. This review highlights the long-term behavioral consequences of adolescent social isolation rearing on the NAc. It will discuss the cellular and molecular changes within the NAc that might underlie the long-term effects on behavior. When available sex-specific effects are discussed. Finally by mining publicly available data we identify, for the first time, key transcriptional profiles induced by adolescence social isolation in genes associated with dopamine receptor 1 and 2 medium spiny neurons and genes associated with cocaine self-administration. Together, this review provides a comprehensive discussion of the wide-ranging long-term impacts of adolescent social isolation on the dopaminergic system from molecules through behavior.

## Introduction

Adolescence is a period of enormous physical, emotional and cognitive growth. It is a highly conserved, necessary developmental stage during which individuals establish independence from their parents (1). From an evolutionary perspective, it is thought that the adolescent period, and the independence established during this developmental period, is crucial for species to reduce the likelihood of incest within a population (2). Adolescent individuals display increased sensation seeking, risk taking and exploration behaviors (3–5), which are thought to be crucial in the specialization of neurobiological pathways in the transition to adulthood (6). Studies using fMRI in humans have shown a peak in reward system sensitivity during adolescence when compared to children and adults (7). Adolescents also show an enhanced vulnerability for psychiatric disorders including anxiety, depression, eating disorders, and substance use disorders (SUD) (8). The prevailing theory of why adolescence is such a crucial period for the emergence of these disorders posits a developmental mismatch between increased striatal-driven sensation-seeking and a lack of prefrontal inhibition of impulsivity (9), or a predominance of reward systems when compared to cognitive control systems (5), which in turn results in individuals who are more sensitive to both stressful and rewarding stimuli (9).

It is well-established that stress sensitivity in adulthood influences susceptibility to depression, anxiety and SUD and the effects of stress are exacerbated when they occur in early life (both gestational and neonatal) (10, 11). Recent evidence suggests that similar to early life, stressful experiences during adolescence may result in long-term, even permanent alterations to brain structure and function (12). Given the developmental emergence of numerous psychiatric disorders associated with adolescence (13–15), research into the neurobiological underpinnings of adolescence will not only contribute to a basic understanding of normative social, emotional, reproductive, and cognitive development, but can further the understanding of health risks and disorders that impact this life stage.

## Adolescence and the Development of Social Reward

Restructuring of social organization is specific to adolescence and is necessary for social species, including both humans and rodents, to develop behavioral strategies that are essential for survival in adulthood. This process is associated with permanent changes in the brain and periphery that are believed to maintain and support the expression of crucial reward-associated behaviors in adult individuals (16). Importantly, disruption of this process may lead to maladaptive behaviors in adulthood (1, 12, 17–20). In humans, adolescent individuals undergo vast changes in social reward and experience a qualitative shift in the salience of social interactions (1). Prior to adolescence, children direct much of their social attention toward their parents and immediate family/caregivers. However, in adolescence, this attention shifts away from parents toward peer-peer interactions (21), with adolescents increasingly relying on their contemporaries for social support and becoming highly sensitive to treatment by their peers (22). Adolescent social experiences have been implicated in the development and maintenance of behavioral pathology into adulthood (23–25).

Rodents display changes in social dynamics that reflect those seen in humans. Many of these changes are sex-specific, with males exhibiting greater sensitivity to social reward than females. In adolescence, male rats show a greater preference for social stimuli in a conditioned place preference (CPP) test when compared to adults (26, 27) or females (26, 28), and males place a greater value on peer-directed activities including play behaviors (29). This enhancement of preference is most pronounced in males deprived of social interactions. Additionally, a peer-paired chamber negates psychostimulant-induced CPP (27, 30) in adolescent males but not females (28).

## Adolescence and Social Stress

Given that adolescent organisms develop heightened sensitivity to stress concurrently with sensitivity to social reward (31), it follows that adolescence would be a period for enhanced sensitivity to social stress specifically. Indeed, disruption of social structures during adolescence has been shown to influence reward, and anxiety- and depression-associated behaviors in rodent models. For example, social instability stress influences depressive-, anxiety-, and reward-associated behaviors (18) receptor binding (32); physiology (33) and gene expression (34) as do many other adolescent stressors including social defeat stress and unpredictable stress models (12, 35–39). Importantly, the effects of social stress during adolescence differ depending on the experimental paradigms but many of these stressors have long-term impacts on behavior and cell function [Review in (40)]. One of the most prominent social stressors is post-weaning social isolation stress, wherein an animal is isolated throughout adolescent development. Though protocol specifics differ, this paradigm involves an animal being placed in a cage by itself throughout the adolescent period. In most paradigms, the timing of isolation coincides with the initiation and enhancement of play behavior (29) and lasts until after play behavior declines in adulthood (41, 42) thus depriving the animal of social interactions during this developmental window.

Generally speaking, adolescent social isolation is thought to be a model of loneliness in rodents and in humans. Loneliness has been identified as a risk factor for various neuropsychiatric disorders (43). This model may be particularly relevant to human health given the social conditions enacted as mitigation for the Covid-19 pandemic—the translational relevancy of this model cannot be overstated. First, lack of direct peer interactions during adolescence has been shown to increase depression like symptoms (44–46). Additionally, recent evidence in humans suggests that adolescent males and females who spend more time on social media are lonelier than their peer counterparts (45). While there are many complicating factors that might influence how social media use impacts loneliness, the data are compelling and suggest that animal models of loneliness might be especially relevant in modeling the emerging changes in social interactions in teenage humans. Additionally, evidence suggests that Covid19 mitigation factors led to significant increases in screen time, usage frequency (47), and increases in social media use (48). Therefore, there is an urgent need to understand the long-term behavioral impacts of adolescent isolation and their underlying cellular and molecular mechanisms.

Post-weaning social isolation stress has been widely used to understand how social experience influences behaviors in adulthood and has yielded conflicting results. These differences are likely due to differences in experimental design (49). Though adolescent social isolation stress paradigms have been widely used, there is currently no standard protocol. The majority of these paradigms involve a period of isolation housing in standard caging beginning around P21–P30 and ending after 3–8 weeks. The timing of isolation is critical for the long-term behavioral effects because of the suite of neural, endocrine, and behavioral changes occurring during adolescence. For example, due to the progression of play behavior across adolescence (42), starting isolation on P21 vs. P28 can lead to critical differences in social reward development. In most cases, animals in social isolation paradigms are not resocialized before behavioral testing (12, 49). This adds a level of uncertainty to the analysis, as it is difficult to determine if the effects are due to social isolation throughout their lives or if the adolescent window is indeed a sensitive period to the effects of adolescent stress. Throughout the review, the term “isolation rearing” will be used when discussing studies that do not resocialize and will be abbreviated as “SI-R.” Those studies that do resocialize are noted and abbreviated as “aSI,” to help clarify effects that may be sensitive to adolescent-specific perturbations. Finally the term “adolescent social isolation” will used as an overarching term to include both stressors (SI-R and aSI) as a way to summarize the general effects of social isolation stress on endpoints. While this is an important distinction, this topic has been extensively covered in another review. For more information regarding the specific effects of isolation rearing vs. adolescent specific isolation please see (50).

## Developmental Changes in the Nucleus Accumbens Occur During Adolescence

**Figure 1:**
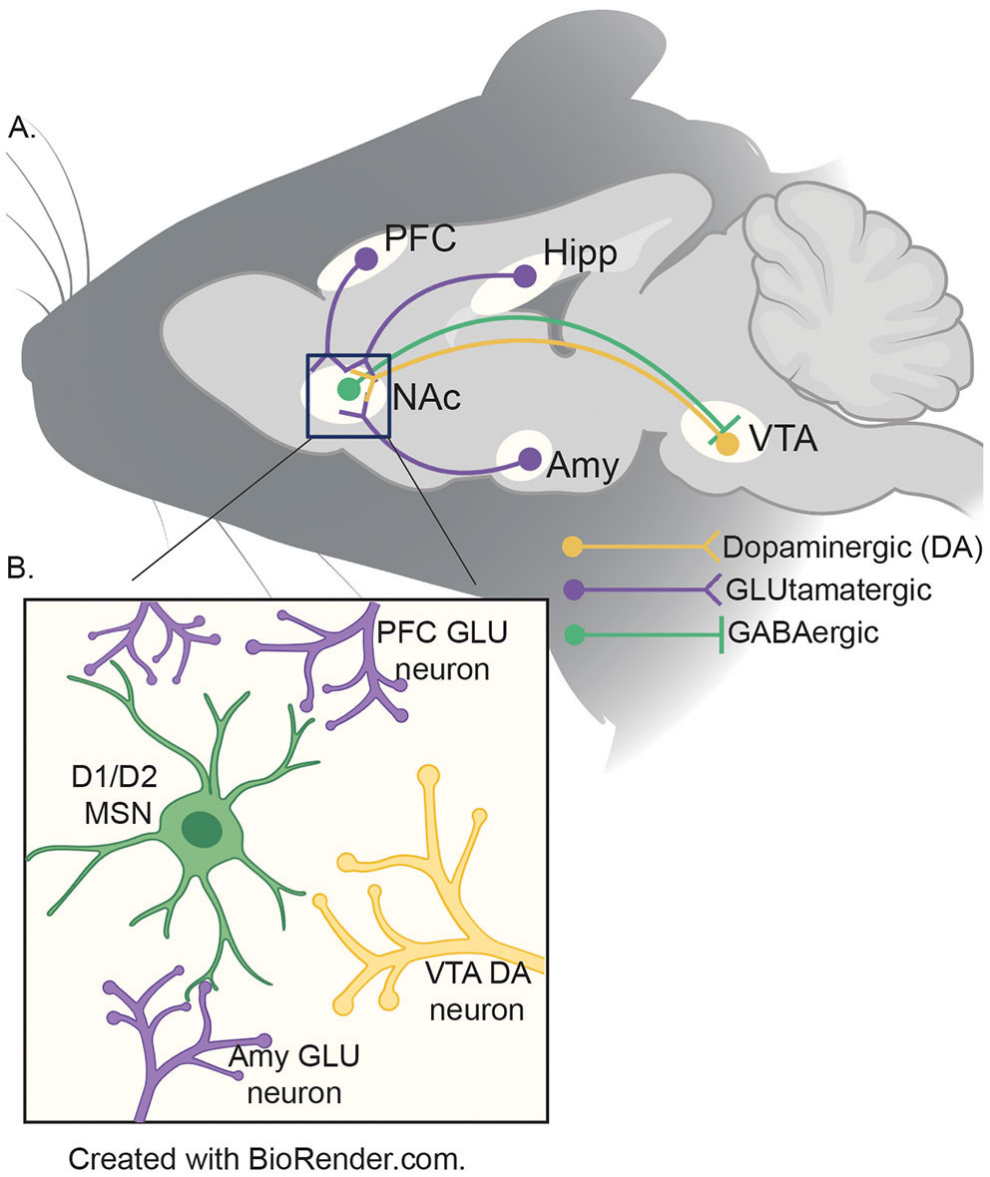
**(A)** Schematic of reward circuitry in rodent brain. The NA receives dopaminergic projections (yellow) from the ventral tegmental area (VTA) and glutamatergic (purple) inputs from the prefrontal cortex (PFC), ventral hippocampus (HIPP) and basolateral amygdala (AMY). GABAergic (green) neurons from the NAc project to the VTA by both direct (D1) and indirect (D2; *via* the ventral pallidum - not shown) pathways. **(B)** Presynaptic inputs to the NAc medium spiny neurons (MSNs). The two major neuronal cell types found in the NAc are D1- and D2-MSNs (green) which integrate information from the VTA (yellow), PFC, HIPP and AMY (purple) to coordinate the appropriate behavioral responses to salient stimuli.

The aforementioned developmental mismatch between sensation-seeking behavior and inhibition is due to divergent developmental trajectories in neural modules mediating these phenomena (9). The NAc is a basal ganglia structure that integrates cortical and limbic information to mediate reward-related behaviors (51). Experience-dependent alterations in the NAc are thought to contribute to the entrenchment of processes fundamental to addiction and vulnerability to stress (52, 53). The NAc is a site of reciprocal projections to and from the VTA, a site of origin of dopaminergic signaling implicated in motivated behavior (Figure 1) (54). The NAc receives dopaminergic modulation from the VTA while receiving glutamatergic input from the various other brain regions in the reward circuitry to include, the prefontal cortex, hippocampus, and the amygdala (55). While more research is necessary, recent evidence suggests that input from the vHIPP to the NAc is a key regulator of the effects of adolescent social isolation (56).

Dopaminergic activity in the NAc is regulated through tonic inhibition by the prefrontal cortex (PFC); reduction in inhibitory tone to NAc leads to hyper responsiveness in the NAc (57). This organization is consequential for reward-seeking during the adolescent period because PFC function is not fully developed until after the pubertal period. This leads to stimuli eliciting high levels of dopaminergic activity in the NAc without inhibition from the PFC (58, 59). Because this circuitry is still developing during adolescence, it is likely that disruptions (e.g., social stress) can have prolonged impacts on its connectivity and function. While other neurotransmitter systems are thought to be almost fully developed prior to the adolescent period, evidence suggests that the dopaminergic system is still developing throughout adolescence (60). For example, Drd1 and Drd2 receptor expression peaks in adolescence (61, 62) and is different between the sexes, with an attenuated peak observed in females when compared to males (63). Importantly, these developmental changes are closely associated with the emergence of sex differences in reward-related behaviors (64–66). Therefore, it follows that stress during adolescence may be especially potent in shaping the developmental trajectory of the reward circuitry, and the dopaminergic system in particular.

The major neuronal cell types in the NAc are medium spiny neurons (MSNs), categorized by the dopamine receptors they express, Drd1 or Drd2. These two subpopulations of MSNs perform different functions in regulating motivated behavior: D1-MSNs convey reward signals and primarily project to the VTA, whereas D2-MSNs encode aversion and primarily project to the ventral pallidum (67–69). This oversimplified separation in classification and function of the major cell types has helped to model MSN function and connectivity in the NAc. However, recent findings have demonstrated overlaps in function and receptor expression of neuronal populations (70–72). Regardless of the specific effects on motivated behaviors, the fact that Drd1 and 2 are expressed in a cell-type specific manner in the NAc suggests that disruptions to dopaminergic signaling by adolescent experience may result in cell-type specific effects and different impacts on behaviors in adulthood.

In an effort to better understand the effects of adolescent social isolation on the NAc we present a comprehensive review of the impacts of isolation on reward-associated behaviors, cellular physiology, gene and protein expression and transcriptional patterns. While ample evidence exists to suggest that SI-R and aSI disrupt anxiety- and stress-related behaviors, it is beyond the scope of this review to include those data. For more information regarding how other behaviors are disrupted by adolescent social stress see (12, 40, 49). Because reward is highly associated with dopamine signaling within the NAc, we have chosen to focus on only those cellular and molecular processes associated with dopaminergic function. For more information regarding how SI-R/aSI influences other neurotransmitter systems see (50).

## Adolescent Social Isolation Stress Disrupts Adult Behavior

As mentioned above, adolescent social isolation (both SI-R and aSI) has been used as a preclinical model of various psychiatric disorders for decades, including models of major depressive disorder, anxiety disorders and SUD, especially in males. All of these behaviors have been linked to disrupted reward processing and are therefore likely influenced by NAc connectivity, activity, and function. Below, we briefly summarize the behavioral impacts of adolescent social isolation specifically on addiction-related behaviors. While it is beyond the scope of the review to go into great detail regarding these findings, more information on the behavioral impacts of adolescent social isolation can be found at (50).

### Addiction-Related Behaviors

Substance use disorder (SUD), is a complex disorder characterized by the cyclical continued use of drugs of abuse despite negative consequences (73). In humans, SUD begins with the escalation of use of drugs of abuse after initial recreational exposures. This is often followed by a cycle of abuse, punctuated by uncontrolled use followed by periods of abstinence or withdrawal and relapse as the condition becomes more severe (74). While vulnerability to SUD appears to have a heritable component [~40%; (75)], substantial evidence suggests that early-life adversity, and adolescent experience in particular, influences susceptibility to addiction in adulthood (76). Although the mechanisms of action between drugs of abuse vary, they all influence mesolimbic reward circuitry to increase the release of dopamine into the NAc. As mentioned, the brain regions that comprise the mesocorticolimbic pathway (Figure 1) are crucial for reward-associated behavior and are still developing during adolescence (77). Therefore, disruption of their developmental trajectory induced by adolescent stress may present as alterations in the reward-associated behaviors regulated by the NAc.

### Conditioned Place Preference

**Figure 2:**
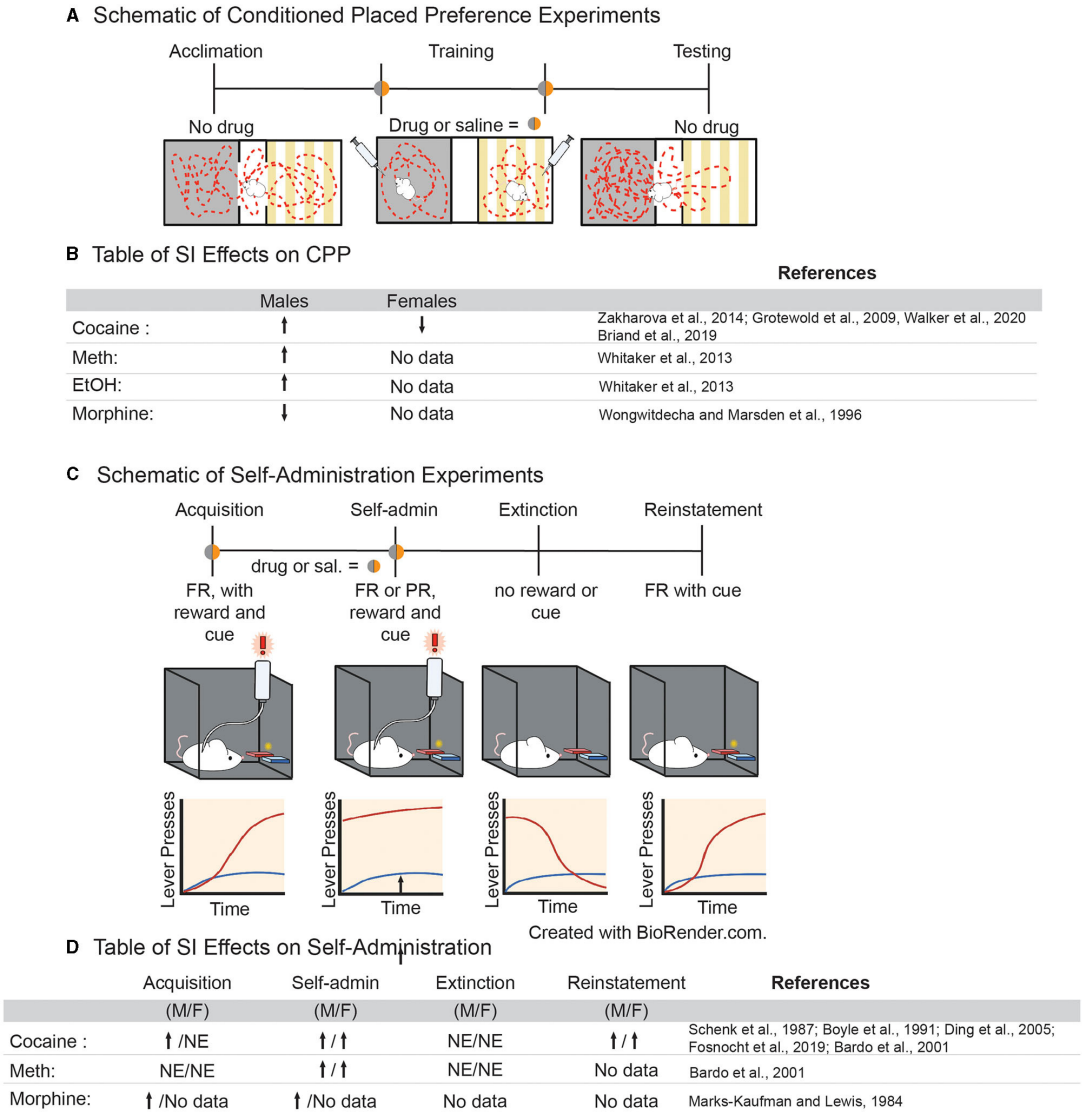
**(A)** Schematic of experimental approach for conditioned place preference paradigms. **(B)** Table of effects of adolescent social isolation/ isolation rearing on CPP behavior in males and females. **(C)** Schematic of experimental approach for volitional self-administration for drugs of abuse in rodents. **(D)** Table of effects of adolescent social isolation on different aspects of self-administration in males and females.

Evidence that adolescent social isolation increases preference for various drugs of abuse in a conditioned place preference (CPP) paradigm is convincing. In this paradigm, an animal is trained to associate a drug of abuse with a chamber that differs in sensory modalities (Figure 2A). If the animal spends more time in the chamber that was paired with the drug when compared to the unpaired chamber, it is said to have formed a preference for the drug experience, which is thought to be indicative of the rewarding properties of a drug (78). Multiple studies have shown that SI-R influences sensitivity to drugs of abuse in CPP, often in a dose-dependent manner [for review, see (50); Figure 2B]. Isolation increases preference for higher doses of both amphetamine (79) and cocaine (80, 81) but not lower doses (82, 83) and only if SI-R began in early adolescent/late juvenile stage (P21) (79). Interestingly, the few studies investigating isolation in females found that SI-R has no effect on cocaine CPP at a low dose [2 mg/kg; (81)]. However, this effect may be dose, species and/or isolation-type specific as others have shown that resocialization after aSI decreases preference for cocaine at a higher dose [7.5 mg/kg; (84)] in mice. Similarly, SI-R increases preference for ethanol in male rats (79). In contrast, SI-R decreases the preference for a low dose of morphine when compared to group house (GH) male rats (85), suggesting that the effects of SI may be specific to substance pharmacology (Figure 2B for Table). These results highlight the need for further research on how adolescent isolation influences the response to different classes of drugs of abuse in both males and females, as well as determine how different neuropharmacological systems (e.g., dopaminergic, GABA, and opioid signaling) develop and are disrupted by adolescent experience.

### Self-Administration

Drug self-administration is a powerful translational model to study SUD. In this paradigm, animals are able to consume drugs voluntarily, which is thought to more accurately represent patterns of drug consumption in humans (78). To accomplish this, animals are often implanted with a jugular vein catheter and learn to activate an instrumental mechanism, e.g., by pressing a lever or nose poke, which then delivers an infusion of drug into their bloodstream (Figure 2C). An animal learns to discriminate between “active” (drug infusion) and “inactive” (no consequence) levers, while the drug serves to reinforce learning the operant task. This paradigm can be divided into stages to model different aspects of SUD (Figure 2C). For example, measuring the time it takes for an animal to discriminate between the active and inactive levers is called “acquisition” and it is thought to model early stages of SUD (Figure 2C). Once animals have acquired operant behavior other variables can be manipulated (discussed in more detail below) to determine various aspects of drug taking behavior which reflect the cycle of SUD in humans.

Generally, variables that increase the rate of acquisition are considered to be risk factors for SUD, while those that delay acquisition are considered to be protective against SUD (78). Studies have shown that both aSI (86) and SI-R increase acquisition of psychostimulants (87–89) and opiates (90) in a dose-dependent manner (Figure 2D). The effects of adolescent social isolation on the acquisition of cocaine self-administration, in particular, have been extensively studied. In males, SI-R increases acquisition at lower doses (between 0.083 and 0.1 mg/kg/inj.), when GH control animals do not acquire, suggesting that SI-R increases cocaine reinforcement at lower doses (87, 89, 91, 92). However, at higher doses (between 0.5 and 1 mg/kg/inj.), the effects of SI-R on acquisition are more varied as studies have shown that acquisition is delayed or not impacted by SI-R (88, 89, 91). Together, these results suggest that SI-R may increase sensitivity to cocaine, meaning that lower doses are more reinforcing but higher doses become aversive. However, the observed effects on cocaine sensitivity may be the effect of prolonged social isolation rather than an adolescent specific effect, as aSI had no effect on the cocaine dose response curve in male rats (86). Interestingly, dose response experiments using amphetamine, another psychostimulant which advances acquisition after SI-R (88), suggests that isolated males are *less* sensitive to amphetamine, as indicated by a rightward shift in the dose response curve (88). Together, these data suggest that adolescent social isolation advances acquisition of self-administration at low doses for various drugs of abuse. However, it is difficult to conclude why, as the findings are somewhat inconsistent and data from dose response curves don't support the hypothesis that drug sensitivity is the driving cause. These results demonstrate a need to standardize protocols for adolescent social isolation, as differences in experimental design may lead to high variability in the study outcomes.

Once an animal has acquired the operant task, lever pressing and intake are measured as an indicator of the reinforcing properties of a drug of abuse (Figure 2C) (78). To our knowledge, no study has investigated how aSI might impact intake. However, SI-R in males generally increases intake of cocaine (87, 93, 94), amphetamine (95), and morphine (90), once again suggesting that social isolation may result in altered sensitivity to drugs of abuse. Similar to acquisition, the effects of cocaine self-administration are dose dependent; no difference is observed in SI-R animals when compared to GH control animals at low doses (0.08–0.32 mg/kg/inj.), but SI-R animals increase intake of cocaine at higher doses (0.5 and 1 mg/kg/inj) compared to GH control animals (87, 93–96). While these dose dependent differences are difficult to interpret, it should be noted that no studies have found that GH control animals consume more drugs of abuse than their SI-R counterparts. Once again, these results should be interpreted with caution, however, as it has been shown SI-R animals press both the active and inactive levers more than control animals (87, 93–95). This may reflect isolation induced hyperactivity which is well-established [reviewed in (97)]. Regardless of the underlying reason for increased lever pressing and intake, it should be noted that this behavioral difference between isolated and control animals will ultimately lead to more exposure to drugs in an SA session for isolated animals and will presumably lead to faster acquisition and greater intake. Finally, it is noteworthy that ADHD/hyperactivity is a risk factor for SUD (98). Thus, adolescent social isolation may provide a translational model for understanding the behavioral, neural and cellular/molecular mechanisms underlying susceptibility to SUD.

While understudied, other behavioral measures of SUD may provide more information regarding how adolescent social isolation may influence the progression of addiction-like behaviors in animal models. Escalation of intake, which models the transition from recreational to compulsive drug taking (78), is increased by isolation (91). Isolation also increases the break-point for cocaine in a progressive ratio paradigm in male rats even after resocialization (86) and in female mice after SI-R (96) suggesting that isolated animals are more motivated to take cocaine than GH controls. Finally, isolation increases lever pressing in a cue-induced reinstatement paradigm, a task associated with relapse, in both male and female mice (96). Together, these data suggest that adolescent SI-R, and to a lesser extent aSI, affects all stages of the addiction cycle.

### Summary of Addiction-Related Behaviors

Together, the current data suggest that social isolation stress affects susceptibility to SUD. Conditioned place preference and self-administration data suggest isolation-induced hyperactivity combined with changes in reward sensitivity may induce susceptibility to addiction in adulthood. However, research practices must be standardized and studies must be expanded to include females, to better assess the underlying reasons for the observed pharmacological differences across studies.

## Adolescent Social Isolation And Disruption Of Cellular Function With in the NAc

There is ample evidence that the reward circuitry, and NAc in particular, regulates reward-associated behaviors (99–103). Stress in adulthood influences synaptic plasticity in the NAc and various stress paradigms have been shown to increase spine complexity and density (104), as well as influence protein and gene expression of molecules associated with synaptogenesis (105). Less is known about how adolescent stress—and isolation stress in particular—influence synaptogenesis within the NAc. One notable study (106) found no change in spine density following SI-R. Although further investigation is needed to elucidate the different mechanisms, these findings suggest that age specific stress-induced structural changes within the NAc may contribute to the long-term behavioral consequences of adolescent social isolation. Furthermore, these plasticity and structural changes following adolescent social isolation and other adolescent stressors are likely associated with changes to neurotransmitter release and uptake as well.

### Adolescent Social Isolation and Dysregulation of Dopamine Signaling Within the NAc

**Figure 3:**
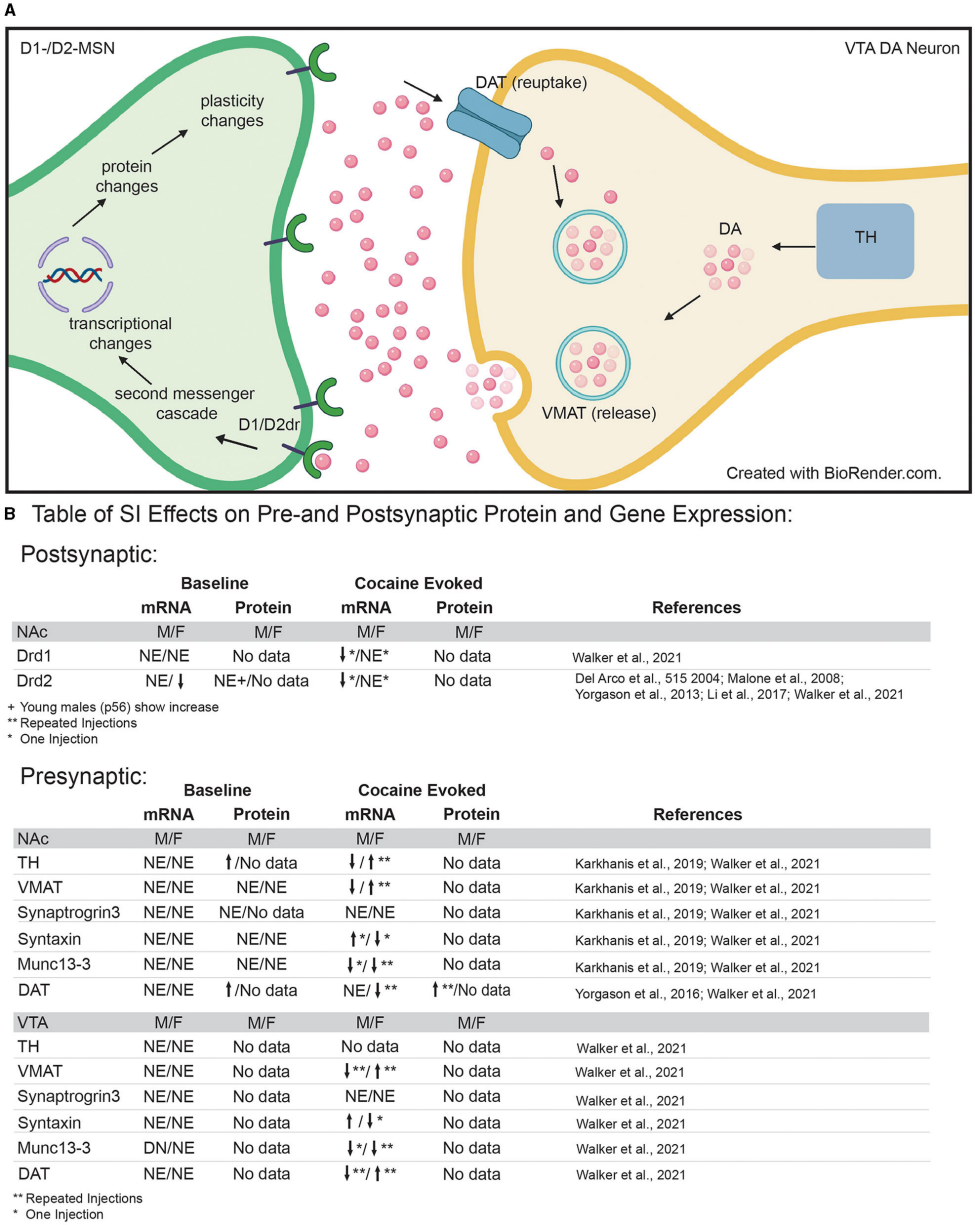
**(A)** Diagram of pre- and post-synaptic mechanisms that may be disrupted by adolescent social isolation. Presynaptic neuron in VTA = yellow; post-synaptic neuron in NAc = green. **(B)** Table of pre- and post-synaptic effects of adolescent social isolation on genes and proteins associated with dopamine synthesis, packaging, release, reuptake and signaling. TH, Tyrosine hydroxylase; VMAT, vesicular monoamine transporter; DAT, dopamine transporter; DA, dopamine molecule; Drd1/2, dopamine receptor 1 and dopamine receptor 2; NE, No Effect; arrows indicate up and down regulation, respectively; *Indicates results after a single injection of cocaine; **indicates results after repeated injections of cocaine.

While numerous neurotransmitters and neuropeptides influence reward integration in the NAc (60, 107), dopaminergic signaling is one of the most well-studied systems with regards to adolescent social isolation (50). Dopamine release into the NAc is a measure of both dopaminergic cell function and responsiveness and SI-R has been shown to affect dopamine release, reuptake and metabolism in reward-associated regions in a region-specific manner [Figure 3; (50)]. With regard to the NAc, SI-R has no effect on extracellular dopamine levels at baseline (89, 108–112), but increases basal dopamine turnover (110, 112), suggesting that SI-R alters baseline dopamine release and uptake mechanisms at least in males.

While baseline levels of dopamine release and reuptake contribute to our understanding of how SI-R might disrupt dopamine neuronal function, studies examining evoked dopamine release provide important information regarding how SI-R might influence the response to aversive and rewarding experiences. These effects are also brain region specific (50) but with regard to the NAc, SI-R impacts both aversive and reward evoked DA release. Once again, to our knowledge, no studies have investigated how aSI specifically affects dopamine dynamics. Therefore, we have only reported SI-R effects here. Generally speaking, SI-R increases aversion- [footshock; (109, 113)] and reward- [drug; (110, 111, 113, 114)] evoked dopamine release into the NAc when compared to GH counterparts, an effect that was also observed after electrical stimulation of dopaminergic neurons (115). These results suggest that dopaminergic neurons are more responsive to stimulus after SI-R. It has been hypothesized that this hyper-responsiveness of the dopamine system may underlie the behavioral consequences of SI-R. In support of this, one study demonstrated that depleting dopamine in the NAc, to bring levels in line with those observed in GH control animals, resulted in a reversal of behavioral phenotypes that usually result after SI-R (116). Combined, these results suggest that isolation rearing may impact dopamine signaling within the NAc through hypersenstivity of dopaminergic neurons to environmental stimuli. This may provide valuable insight in to the mechanisms regulating the behavioral effects of SI-R on aversion- and reward-related behaviors. Further research is necessary to better understand how SI-R affects the readily available pools of dopamine, and how generalized the stimulus-induced increase in dopamine released into the NAc may be (e.g., social stimuli). This would be helpful in broadening our understanding of what evoked dopamine release is encoding within the NAc and how adolescent experience influences these developmental parameters. Finally, given the sex differences in reward processing more research is necessary to better understand how adolescent experience might influence dopamine release in females.

## Adolescent Social Isolation Impacts on Dopaminergic Protein and Gene Expression in the NAc

The findings above indicate that both SI-R and aSI induce behavioral phenotypes that may be sex-specific and are associated with cellular changes within the NAc. Because cellular physiology is ultimately the result of differences in gene/protein expression and function, several studies have sought to understand how adolescent social isolation in general impacts expression within the NAc. Several families of genes and proteins have been studied after adolescent social isolation throughout the brain including growth factors (117) and glutamatergic signaling (118). However, it is beyond the scope of this review to cover all expression changes observed after adolescent social isolation. Therefore, we will primarily focus on those molecules associated with dopamine production, release, and signaling. For more information on how adolescent social isolation impacts other protein families see (117).

When dopamine is released from the VTA dopaminergic neurons, it binds to its receptors on the cell surface of the D1- and D2-MSNs in the NAc which, in turn, activates second-messenger signaling cascades, ultimately leading to changes in transcription of RNA and the translation of new proteins (Figure 3). DA receptors differ in the second-messenger cascades activated by binding and display differences in affinity for DA (119). Therefore, differences in expression and activation of dopamine receptors within the NAc will result in differences in gene/protein expression within these neurons and ultimately lead to changes in synaptic plasticity and spine formation between neurons within the NAc and between other brain areas. This interplay between second-messenger signaling, synaptic changes, and cellular connectivity highlights the dynamic nature of reward processing within the NAc. Additionally, the aforementioned developmental changes in dopamine receptor protein expression and receptor binding (62) in the NAc suggest that stress during adolescence could disrupt the developmental trajectory of dopamine receptor expression. This would influence both pre- and post-synaptic expression in dopaminergic neurons and their afferents, thus influencing downstream behavioral outcomes.

### Adolescent Social Isolation Impacts on Presynaptic Dopaminergic Genes and Proteins

Given that adolescent SI-R alters reward and aversion evoked release of dopamine in the NAc (see references above), it follows that presynaptic mechanisms regulating dopamine release into the synaptic cleft may be altered by adolescent social isolation. To gain a better understanding of how adolescent social isolation might influence dopamine signaling, studies have focused on changes in expression of genes/proteins associated with production, packaging, release, and reuptake of dopamine (Figure 3B). Regarding dopamine synthesis, studies have investigated how tyrosine hydroxylase (TH), the rate-limiting enzyme in dopamine synthesis, expression is impacted by adolescent social isolation. Changes in its expression are often interpreted as a reflection of the dopamine available for release. In rats, SI-R increases TH protein-expression in synaptoneurosomes from the NAc when compared to GH males (120), which could explain the increased evoked dopamine observed after SI-R discussed above (section Adolescent Social Isolation and Disruption of Cellular Function With in the NAc). However, these effects may be species, molecule, and/or isolation type specific as a recent study investigating gene expression changes in mice (121), found that *Th* mRNA expression was unaffected by aSI in males and females in both the NAc and VTA. Additionally, important sex-specific changes in *Th* mRNA expression in the VTA, the presumed location of Th mRNA transcription, were observed after exposure to cocaine (Figure 3B). When animals were exposed to repeated cocaine injections (10 days, 7.5 mg/kg), aSI males and females display opposite regulation of *Th* expression: females upregulate and males downregulate *Th* expression when compared to their same-sex GH counterparts. Additionally, aSI males but not females downregulated *Th* gene expression after only one injection of cocaine (121). Together, these data suggest that adolescent social isolation alters the synthesis of dopamine in a sex, species and stimulus-specific manner. While further research is necessary, these data provide intriguing evidence that differences in TH gene and protein expression may lead to altered levels of presynaptic dopamine available for release after adolescent social isolation.

Once dopamine is synthesized, it is packaged into vesicles for release. Vesicular monoamine transporter (VMAT) is the protein necessary for packaging dopamine into vesicles (Figure 3A). Studies investigating VMAT protein levels (120) and RNA levels (121) found that adolescent social isolation has no effect on expression in the NAc of adult male rats (120) or male and female mice (121). However, aSI induced sex-specific changes in gene expression of VMAT (gene name: *Slc18a2*) in the VTA (Figure 3B), the presumed source of VMAT protein in terminals of dopaminergic neurons (Figure 3A). While *Slc18a2* expression was unaffected at baseline, when animals were exposed to repeated cocaine injections (7.5 mg/kg for 10 days), sex-specific regulation of expression was again observed: females upregulated whereas males downregulated *Slc18a2* expression [Figure 3B; (121)]. These data suggest that baseline packaging of dopamine into vesicles is unaffected by adolescent social isolation. However, aSI (SI-R not studied) may disrupt the response to repeated cocaine in a sex-specific manner and further studies should focus on how dopamine packaging may be disrupted by adolescent social isolation after cocaine exposure.

Release of dopamine occurs when vesicles dock with the cell membrane and release dopamine into the synaptic cleft. Kharkanis et al. (120) investigated changes in proteins involved in exocytotic dopamine release, synaptic priming, and vesicular docking after SI-R and found no significant expression changes in synaptogrin-3 (Syngr3), syntaxin-1 (Stx1a), and Munc13-3 (Unc13c) in male rats. Similar effects were observed after aSI on baseline mRNA expression in male and female mice. With the exception of *Unc13c* mRNA expression in the VTA where aSI males had less *Unc13c*, aSI had no effect on baseline gene expression of these molecules in the NAc or VTA (121). However, cocaine evoked changes in mRNA expression were observed across both brain regions and sexes. While, aSI had no effect on cocaine induced changes in *synaptogrin-3* (Syngr3) mRNA, opposite regulation of *syntaxin-1* (Stx1a) mRNA in males and females was observed after SI: aSI females downregulated and aSI males upregulated *syntaxin-1* mRNA in response to the first dose of cocaine (121). Regarding *Unc13c* mRNA expression in the NAc, sex-specific regulation in response to cocaine was also observed: aSI females downregulated *Unc13c* in response to repeated cocaine injections whereas aSI males downregulated *Unc13c* in response to the first dose of cocaine (Figure 3B for Table). These differences point to important cocaine-induced sex-specific transcriptional effects evoked by aSI that may point to the regulatory mechanisms underlying observed sex-specific reward-associated behavioral effects. Together, these data provide intriguing evidence that the evoked changes in dopamine release by drugs of abuse may be the result of changes in vesicular docking.

Finally, after release into the synapse, dopamine is taken back up into the presynaptic cells through the dopamine transporter (protein = DAT; gene = *Slc6a3*) (Figure 3A). Pharmacologically, cocaine and amphetamine increase available dopamine levels in the synapse by blocking DAT function, thus preventing dopamine reuptake into the presynaptic cell for rerelease. Previous studies have shown that DAT protein expression is increased in the NAc after SI-R (114) which is further induced by 3 daily injections of cocaine in SI-R animals. Importantly, this effect on cocaine induced DAT expression was blocked by rearing animals in an enriched environment (80). Recently Walker et al. (121), investigated gene expression changes of *Slc6a3 mRNA* in the VTA of mice using RNA-sequencing. In contrast to protein levels, no baseline differences were observed. Again, these differences may reflect differences between species (rats vs. mice), isolation type [SI-R (114) vs. aSI (121)], or molecule investigated (protein vs. mRNA). Once again, cocaine induced sex-specific regulation of Slc6a3 mRNA: aSI females downregulated *Slc6a3* in response to repeated cocaine injections (10 days of 7.5 mg/kg), whereas aSI males expression levels were unchanged (121). Importantly, Slc6a3 mRNA expression was altered by repeated cocaine injections in the VTA of both sexes where aSI females upregulated and aSI males downregulated mRNA expression. As previously mentioned, the VTA would presumably be the site of most *Slc6a3* transcription (Figure 3B). Together, these changes in DAT protein and gene expression suggest that adolescence experience may influence dopamine reuptake. Thus, providing a potential mechanism for the altered dopamine dynamics observed in the NAc after adolescent social isolation.

Overall, the fact that gene expression changes in presynaptic molecules were observed in the VTA but few protein expression changes were observed in the NAc suggests that changes may be specific to synapses, and some effects may be washed out by the heterogeneity of the tissue. However, another interpretation of these data could be that adolescent social isolation alters presynaptic proteins in projections from the VTA to other brain regions receiving dopaminergic inputs. Further research is necessary to fully understand how adolescent social isolation disrupts projection-specific signaling within the brain. It is also notable that many of the changes are sex-specific, again highlighting the need for further investigation of the impacts of adolescent social stress in both males and females.

### Adolescent Social Isolation Alters Post-synaptic Dopaminergic Genes and Proteins

The finding that adolescent social isolation influences dopamine dynamics within the NAc suggests that both pre- and post-synaptic dopamine signaling may be altered by adolescent social isolation. Changes in dopamine receptor mRNA and protein expression are indirect measures of post-synaptic dopamine dynamics. To our knowledge, no study has investigated how SI-R or aSI specifically affects Drd1 protein expression or activity. However, RNA sequencing in the NAc showed that aSI has no effect on *Drd1* mRNA expression in male or female mice at baseline, but, aSI males decreased expression after a single dose of cocaine [Figure 3B; (121)]. On the other hand, several studies have found that SI-R has no effect on *Drd2* mRNA, protein, or activity levels in the NAc of adult male rats (115, 122, 123) and adult male mice [aSI; (121)]. However, these effects may be age-specific, as a recent study in younger male mice (~P56) found that *Drd2* mRNA and protein levels are increased in the NAc after SI-R from P21 - ~P56 (124). In females, RNA-sequencing revealed that aSI animals express less *Drd2* mRNA than their GH control counterparts at baseline. As with *Drd1* mRNA, SI males decrease *Drd2* mRNA expression after a single dose of cocaine but no changes were observed after repeated injections of cocaine [Figure 3B; (121)].

Together, these data suggest that adolescent social isolation may impact dopamine signaling through reprogramming expression of dopamine receptors within the NAc in a sex-specific manner. While more research is necessary, the finding that only SI males display cocaine induced changes in expression of dopamine receptors may illuminate the mechanisms regulating the sex-specific behavioral impacts of adolescent social isolation and once again highlights the need to include females in future research.

#### Cell-Type Specific Effects of Adolescent Social Isolation

**Figure 4:**
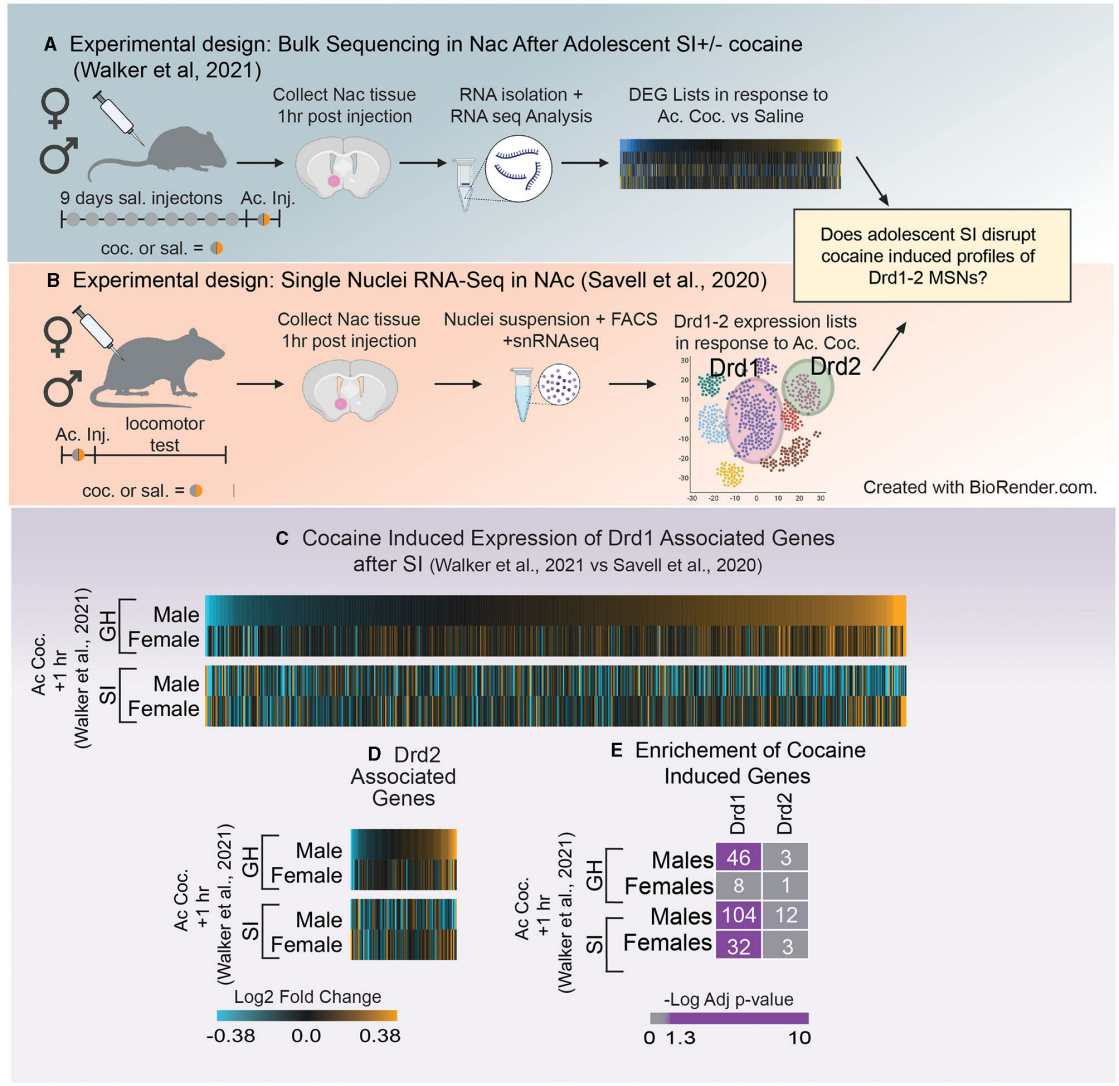
**(A)** Schematic of experimental design utilized in Walker et al. (121) which investigated how adolescent social isolation disrupts the transcriptomic effects of cocaine in males and females. **(B)** Schematic of experimental design utilized in Schematic of Savell et al. (125) which used single nuclei RNA-seq to determine transcriptional profiles nuclei RNA-seq to determine transcriptional profiles of Drd1 and Drd2 MSNs. **(C,D)** Heatmaps of expression of Drd1 associated genes **(C)** and Drd2 associated genes **(D)** after the first dose of cocaine in group housed (GH) and socially isolation (SI) males and females. Unlike GH males SI males downregulate expression of Drd1 and 2 associated genes after a first dose of cocaine. **(E)** Enrichment of Drd1 and Drd2 associated genes and those altered by a single dose of cocaine in GH and SI males and females. GH males but not females regulate Drd1 associated genes in response to the first dose of cocaine. In SI animals, both males and females regulated Drd1 associated genes. Purple = significant enrichment, gray = non-significant enrichment; numbers in boxes = number of genes overlapping between the two lists.

Many of the studies referenced above used a candidate gene/protein approach to identify how SI-R impacts expression of molecules of interest based o*n a priori* hypotheses. Because neurochemistry and electrophysiological studies have identified the dopaminergic system as sensitive to adolescent social isolation, many hypothesized that genes and proteins associated with dopamine signaling would also be impacted. However, few studies have investigated the question of how adolescent social isolation impacts transcription in an unbiased approach. Recently, Walker et al. (121) used RNA-sequencing to investigate the long-term transcriptional impacts of aSI in the NAc of male and female mice (for experimental paradigm see Figure 4A). Through an integration of multiple bioinformatic analyses, the authors not only identified broad patterns of gene expression that were disrupted by aSI but also found essential biological pathways and potential key driver genes that may regulate the aSI-induced transcriptional patterns in male and female mice. Key driver genes are highly connected “hubs” within a co-expression module, a group of genes whose expression changes together across multiple variables. The key driver genes identified in the Walker et al. (121) ranged in biological functions and display sex-specific expression across multiple treatments groups. A full list of key driver genes identified in the PFC, NAc and VTA is published in Walker et al. (84). Overall, the authors observed a greater transcriptional response to acute and chronic cocaine in the NAc of aSI males but not females, with very little overlap in the cocaine-induced differentially expressed genes (DEGs). In this study DEGs were identified using the following thresholds: nominal *p*-value < 0.05 and a fold change of expression of at least 30%. A nominal *p*-value was chosen for the DEG cut-off because the authors were interested in identifying broad transcriptional patterns important for regulating complex behavioral phenotypes. Importantly, this approach, in combination with other bioinformatic analysis, has been applied successfully to other bulk sequencing studies to illuminate functional mechanisms underlying various neuropsychiatric disorders (126–130). As mentioned above, bulk sequencing measures gene expression changes in a heterogenous mixture of cells. This is an important point to consider when making decisions about *p*-value cut offs as transcripts that survive a conservative *p*-value correction are likely reflective of expression changes which occur in the same direction across a number of cell types and may not be as biologically relevant when trying to understand transcriptional regulation behavior. In support of this, Walker et al. (121) replicated decades of studies using candidate gene approaches to identify gene expression changes in response to cocaine in their sequencing datasets. Importantly, in many cases, those changes did not survive a corrected *p*-value suggesting that a corrected *p*-value may be too conservative when applied to bulk sequencing data sets. In addition, pathway analysis revealed that DEGs altered by acute cocaine were associated with dopaminergic signaling in aSI males but not females, suggesting that cocaine-induced transcriptional signaling in D1- and/or D2-MSNs may be disrupted by aSI in a sex-specific manner (121). Recent advances in RNA-sequencing approaches allow us to address this hypothesis here.

The advent of RNA-sequencing provides a unique, unbiased opportunity to analyze the global transcriptome. There are numerous sequencing methods that can be applied to answer questions about how gene expression changes within a specific brain region. Bulk RNA-sequencing, a technique used to measure transcriptomic changes in a homogenized tissue sample, provides important information about all expression changes within a sample, independent of the composition of the tissue. While this approach is informative, the heterogeneous nature of the brain makes it difficult to interpret how specific cells (e.g., D1- vs. D2-MSNs) may be impacted by aSI. However, the advent of single-cell RNA sequencing has led to the ability to determine which genes are representative of specific cell types within a brain region. Therefore, we leveraged two unbiased sequencing experiments to better understand how gene expression patterns within D1- and D2-MSNs might be influenced by aSI. The first measured expression changes induced by aSI using bulk RNA-sequencing data [Figure 4A; (121)]. The second used single nuclei RNA sequencing to characterize transcriptomic profiles in D1- and D2-MSNs after one dose of cocaine in male and female rats [Figure 4B; (125)].

First, D1- and D2-MSN associated gene lists were extracted from Savell et al. (125), using the following parameters (adjusted *p*-value < 0.1). While this study was conducted in rats, we chose this cell-type specific list because it is the first to look at cell-type specific clusters 1 h after an injection of cocaine in both males and females, making it an ideal comparison to the sequencing data in Walker et al. (121), as those animals were also euthanized 1 h after the first dose of cocaine (Figures 4A,B). Importantly, while these gene lists were identified in a different species, we found that ~80% of D1- and 93% of D2-MSN associated genes from rats were identified in the mouse transcriptome. Heatmaps were generated using the Log_2_Fold change in expression (acute cocaine vs. saline) in male and female mice after GH or aSI (Figures 4C,D). For all heatmaps, genes were sorted by expression in GH males. We found that unlike GH males, aSI males downregulated genes assigned as *Drd1* or *Drd2* expressing cells. Additionally, both GH males and females upregulated more *Drd1* and *Drd2* associated genes in response to the first dose of cocaine (~60% upregulated vs. ~40% down) and this pattern was flipped after aSI in males but not females. These data suggest that aSI disrupts cocaine-induced dopaminergic transcription in males.

Next, because the data presented in the heatmaps (Figures 4C,D) do not apply expression or *p*-value thresholds to expression from Walker et al. (121), we next used Fisher's exact testing (131) to determine if genes affected by the first dose of cocaine in GH and aSI males and females (*p*-value < 0.05 and a Fold-Change in express >30%) are enriched in genes associated with D1- or D2-MSNs (Figure 4E). Interestingly, we observed a sex difference in the enrichment of Drd1 associated genes in GH animals but not aSI animals. Genes regulated by cocaine are enriched in Drd1 associated genes in GH males but not GH females, whereas enrichment was observed in both aSI males and females. While the majority of effects of adolescent social isolation have been identified in males, these data provide compelling evidence that aSI may also influence the Drd1 associated transcriptional response to cocaine in females and points to another potential mechanism by which aSI may influence reward-associated behaviors in a sex-specific manner.

### Sequencing Data Reveals Genes Associated With Addiction-Related Behaviors Are Disrupted by Adolescent Social Isolation

**Figure 5:**
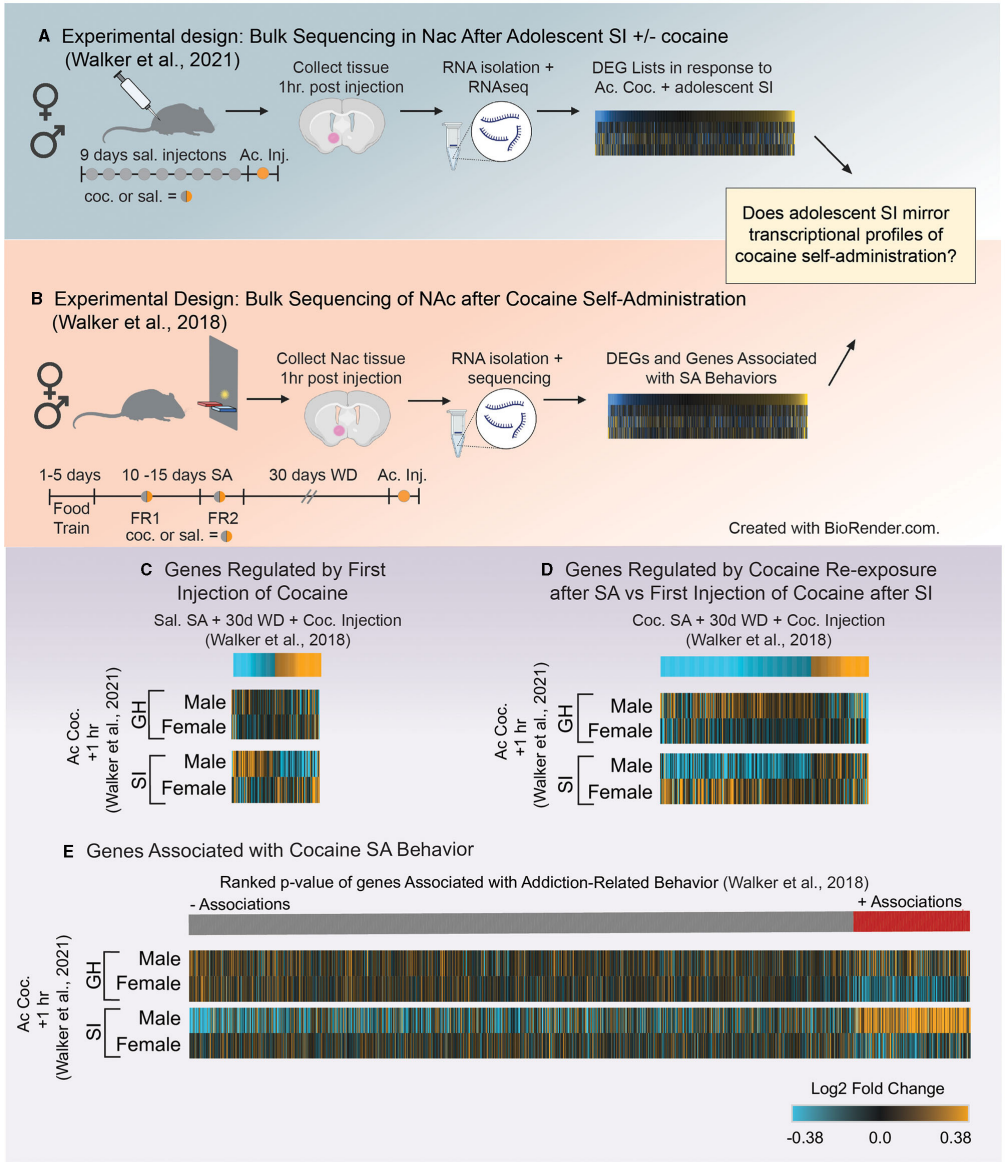
**(A)** Schematic of experimental design utilized in Walker et al. (121) which investigated how adolescent social isolation disrupts the transcriptomic effects of cocaine in males and females. **(B)** Schematic of experimental design investigating transcriptional profiles in the NAc after cocaine self-administration from Walker et al. (132). **(C,D)** Heatmaps of genes altered by different exposure conditions after self-administration (132) compared to expression profiles in group housed (GH) and socially isolated (SI) animals after the first dose of cocaine (121). Expression of genes regulate by the first dose of cocaine after saline self-admin + 30 days of withdrawal (WD) are opposite to those of socially isolated (SI) males after their first dose of cocaine **(C)**. However, transcriptional profiles of genes regulate by the cocaine re-exposure after cocaine self-admin + 30 days of withdrawal (WD) are reflected in the expression profiles of SI males after their first dose of cocaine **(D)** suggesting that SI reprograms the transcriptional response to and initial dose of cocaine to be similar to that of an animal previously exposed to cocaine. **(E)** Comparison of genes associated with self-administration behavior and expression profiles of those genes after the first dose of cocaine in GH and SI animals. Associations of genes with a composite index (addition index) of self-administration behavior are organized by their ranked *p*-values and assigned a color based on the direction of the association (red = positive; gray = negative). Heatmaps of Log2Fold changes in expression in GH and SI animals after the first dose of cocaine are aligned to the associations from Walker et al. (132) to determine if direction of changes in expression are reflected in behavioral associations. Unlike other groups, expression profiles in SI males after the first dose of cocaine reflect the associations of genes related to the addiction index, suggesting that SI reprograms expression of genes related to self-administration behaviors.

The finding that dopaminergic transcription might be disrupted by aSI suggests that transcriptional changes may be an important regulator of reward-associated behaviors. One challenge of transcriptome-wide analysis is identifying genes or patterns of genes associated with behaviors in a statistically valid manner. However, a recent study (132) utilized a number of bioinformatic approaches coupled with exploratory factor analysis to identify patterns of genes that were associated with self-administration and behavior in mice (132). While the studies above suggest that a wide-range of cellular molecular processes are disrupted by aSI, the transcriptional modifications underlying the behavioral differences in aSI animals remains unanswered. In order to better understand how transcriptional changes induced by aSI may be related to long-term behavioral outcomes, we compared transcriptional patterns from two publicly available bulk sequencing datasets. The first analyzed cocaine-induced gene expression changes after aSI in males and females [for experimental paradigm see Figure 5A; (121)]. The second identified DEGs in mice who underwent cocaine self-administration + withdrawal and re-exposure to cocaine. (For specifics regarding experimental design, see Figure 5B). By comparing expression patterns across these two studies, we are able to identify directional changes in gene expression induced by aSI that are reflected in expression patterns associated with cocaine self-administration. Because we are comparing expression changes in two different sequencing studies, it is important to point out that we are only comparing directional changes between the two studies and not overlap of DEG lists, as specific lists of DEGs may be more sensitive to batch effects and differences in RNA-sequencing analytical approaches.

Gene lists were extracted from Walker et al. (132) using the following parameters (*p* < 0.05 and Fold Change in expression>30%; see above for justification) for each comparison [saline SA + 30 days withdrawal + cocaine injection (Figure 5C) or cocaine SA + 30 days withdrawal + cocaine injection; Figure 5D]. We chose these two comparisons from Walker et al. (132) in order to determine if the first dose of cocaine in aSI animals: (1) mirrors expression changes in genes regulated by the first dose of cocaine (Figure 5C) or (2) mirrors expression changes induced by cocaine SA + cocaine re-exposure (Figure 5D). Heatmaps of gene expression changes comparing expression patterns in 132 (Figure 5C, top) to those in Walker et al. (121) (Figure 5C, bottom) revealed that aSI males display opposite patterns of expression in genes regulated by the first dose of cocaine after saline SA (Figure 5C). However, expression patterns of genes that are up- and downregulated by cocaine re-exposure after cocaine SA + 30 days withdrawal (Figure 5D, top) are closely reflected in aSI males after their first *exposure* to cocaine (Figure 5D, bottom). These data suggest that aSI influences cocaine-responsive transcripts to be sensitive to the first exposure to cocaine and that expression patterns closely reflects the pattern of expression induced by cocaine re-exposure in an animal with a history of cocaine SA (Figure 5D). Given the increased susceptibility to cocaine-associated behaviors in males after adolescent social isolation, including cocaine self-administration, these data provide intriguing evidence that a suite of cocaine-sensitive genes may play a role in experience-induced susceptibility to SUD.

As mentioned, Walker et al. (132) leveraged exploratory factor analysis to identify components of self-administration behavior reflective of different stages of self-administration paradigms (Figure 2C). A composite score was calculated for each individual in the study as an “addiction index.” The addition index included values from 3 factors identified using exploratory factor analysis. The first was a measure of an animal's intake during self-administration. In this factor animals who self-administered cocaine had greater intake than animals administering saline. The second was a measure of an animal's ability to discriminate between the active and inactive levers. The third was a measure of increased lever pressing under increased demand (i.e., switching from FR1 to FR2). Each animal's data was combined across these three factors to yield the “addiction index” for each individual. This means that animals that performed well on multiple aspects of the operant tasks (i.e., all three factors) had a high addiction index whereas those that performed poorly had a low addiction index, resulting in a continuous value for each individual which could be associated with gene expression. To our knowledge, Walker et al. (132) was the first study to combine factor analysis of self-administration behavior with transcriptome-wide data to identify genes associated with behaviors in an unbiased approach. Genes that are positively related to behavior are genes that increase with the addiction index whereas genes that are negatively associated with behavior are genes that decrease as the addiction index increases.

Here, we compared the expression patterns in GH and aSI males and females after the first dose of cocaine with those genes positively or negatively associated with self-administration behaviors. Heatmaps are seeded by the ranked *p*-values of the associations (slope > 0.2 and *p*-value < 0.05) from Walker et al. (132) (Figure 5E, top) and reveal that expression profiles of genes after the first dose of cocaine in aSI males reflect the associations with self-administration behavior (i.e., genes that are upregulated by the first dose of cocaine in aSI males are positively associated with the addiction index, and genes that are downregulated by the first dose of cocaine is aSI males are negatively associated with the addiction index). These data suggest that the expression patterns observed in aSI males in the NAc after the first dose of cocaine are similar to those in genes associated with cocaine self-administration behaviors. While further research is necessary, these data once again provide compelling evidence that aSI in males influences the transcriptome in the NAc to be especially sensitive to the first dose of cocaine. Furthermore, while these data should be considered preliminary, they provide potential targets for follow-up to better understand how adolescent experience might influence susceptibility to SUD through transcriptional reprogramming.

### Summary of Molecular Changes Induced by Adolescent Social Isolation

In comparison to the behavioral and circuitry studies focusing on adolescent social isolation and the NAc, the molecular level work is lacking. However, there have been very recent studies as described above focusing on the mechanisms responsible for the widely accepted disruptions adolescent social isolation has on the NAc. Together the data suggest that gene and protein expression changes induced by adolescent social isolation stress are sex-, species-, isolation type, and stimulus-specific. While more research is necessary, these data also suggest that genes and proteins associated with dopaminergic synthesis, packaging, release, metabolism and signaling are all impacted by adolescent social isolation. Future studies looking into the transcriptome and proteome level in a region- and sex-specific manner within the reward system would be crucial in advancements in therapeutic-level interventions surrounding addicted phenotypes.

## Summary and Conclusions

We are only beginning to understand the complex interplay of the neural networks, endocrine signaling, and environmental inputs that drive normative adolescent development. Successful expression of adult-typical behaviors is dependent on a high degree of coordination between these factors during adolescence. Adolescent social isolation disrupts key elements of this coordination, leading to potential pathological alterations in behavior and their molecular and circuit foundations. This review has highlighted some of these alterations and points to a few emerging themes throughout: (1) Adolescent social isolation augments reward-associated behaviors in adulthood, particularly in males, with many studies pointing to differences in altered sensitivity to drugs of abuse. (2) Changes in the dopaminergic system—both at baseline and in drug evoked responses—appear to underlie the reward-associated behavioral consequences of isolation rearing, at least in males (Figure 2). (3) These changes are likely due to modifications to dopamine-associated gene transcription and protein expression, specifically related to dopamine synthesis, packaging, release and receptors, many of which show sex-specific changes in response to cocaine (Figure 3). (4) Comparisons of bulk sequencing after aSI with single nuclei RNA-seq after cocaine reveal that aSI induces vastly different cocaine-induced transcriptional patterns in D1- and D2-MSN associated transcripts, specifically in males when compared to GH controls (Figure 4). (5) By comparing transcriptome-wide datasets, we identified suites of genes with expression patterns after aSI which reflect those observed in animals with a history of cocaine SA, and identify potential transcripts that may be important for experienced-induced vulnerability to SUD (Figure 5). While these themes point to disruption of multiple facets of DA signaling by both SI-R and aSI, further research is needed to better understand the cellular molecular processes underlying the long-term behavioral consequences of adolescent social stress as inconsistencies in experimental design and the lack of inclusion of females subjects makes drawing firm conclusions difficult. Future work needs to standardize and refine experimental design to account for inconsistencies and should include explorations of sex-specific responses to adolescent social isolation. Finally, the Covid-19 pandemic, which has led to radical entrenchments of isolating social conditions for adolescents makes understanding the long-term changes—from molecular to behavioral—all the more urgent. Further investigations will yield critical information related to understanding the psychiatric disorders that emerge during adolescence, and may lead to novel therapeutics and interventions in vulnerable populations.

## Author Contributions

CB, AM, MA, and DW wrote and edited the manuscript. UD helped analyze data. All authors contributed to the article and approved the submitted version.

## Funding

Funding for this work was provided by NIDA (R00DA042100) and the Collin's Medical Trust.

## Conflict of Interest

The authors declare that the research was conducted in the absence of any commercial or financial relationships that could be construed as a potential conflict of interest.

## Publisher's Note

All claims expressed in this article are solely those of the authors and do not necessarily represent those of their affiliated organizations, or those of the publisher, the editors and the reviewers. Any product that may be evaluated in this article, or claim that may be made by its manufacturer, is not guaranteed or endorsed by the publisher.
